# Reliability of Wassermann Test

**Published:** 1915-11

**Authors:** 


**Reliability of Wassermann Test.** O. D. Phelps of Worcester, Boston M. & S. Jour., Sept. 9, sent 113 samples of blood to 4 laboratories with 68 agreements (60.2%); 135 to 3 laboratories with 70 agreements (51.9%); 110 to 2 laboratories with 72 agreements (65.5%). He quotes Craig as stating that the only diseases giving the Wassermann beside syphilis are yaws, leprosy, relapsing fever and malaria in the febrile stage. He considers a repeated positive test as reliable but does not consider a negative test as necessarily excluding the disease. In well marked syphilis (macroscopic appearance, yielding to salvarsan and mercury) alcoholic indulgence may render the Wassermann reaction negative, a positive reaction occurring after the alcohol is discontinued. (Note: In repeatedly publishing abstracts showing marked discrepancies in the Wassermann test, we are not manifesting a spirit of partisanship. None of the recent statistic reports that have come under observation have shown absolute agreement. We have called attention to the fact that a sign or symptom of any kind, to have much value, must show a very small percentage of error, since 50% of occurrence or non-occurrence under ordinary condition means a zero of significance. We have also called attention to the fact that, in most instances, so-called specific reactions show errors in both positive and negative directions and of approximately equal degree. This does not indicate any underlying principle but is merely an empiric observation. A priori, we would expect that a positive demonstration of a germ or of a reaction due to its presence, would be reliable and not subject to error except in confusion of specimens or gross incompetence in applying the test. On the other hand, a negative result—for instance in the search for tubercle bacilli—does not necessarily imply the absence of the disease in question. Of course, two or more germs or reactions due to them, may give similar results—as in the confusion of certain acid-fast bacilli with tubercle bacilli—and in the occurrence of a positive Wassermann in the conditions listed by Craig. Of these yaws is regarded by many as a typic syphilis while leprosy and relapsing fever and, in many parts of America, malarial fever are so rare as to be of little practical importance. Just how much of the unreliability of the Wassermann noted by Phelps, Wolbarst and others is due to incompetence, how much is due to present lack of recognition of factors in technic required to render the test strictly specific and how much is due to a lack of real specificity in the Wassermann reaction even in a perfected technic, can not be stated at present. It is of practical importance, however, to realize that, under existing conditions, a negative Wassermann is significant only to the extent that a negative search for tubercle bacilli is significant and that a positive reaction has just about the same value as a purely clinical opinion of a practicing physician of the same degree of expertness as the corresponding laboratory worker. This latter statement does not mean that a positive Wassermann is unreliable but rather that both laboratory and clinical tests are good. This fact should be borne in mind for medicolegal purposes also. In particular, we feel that the time is not ripe for legislation requiring applicants for marriage to submit to a Wassermann test. Such legislation, we understand, actually exists in some states.

